# The *TP53* Arg72Pro polymorphism predicts visual and neurodegenerative outcomes in retinal detachment

**DOI:** 10.1038/s41419-025-07739-1

**Published:** 2025-05-26

**Authors:** Nadia Galindo-Cabello, Eva M. Sobas-Abad, Rebeca Lapresa, Jesús Agulla, Ángeles Almeida, Antonio López, José Carlos Pastor, Salvador Pastor-Idoate, Ricardo Usategui-Martín

**Affiliations:** 1 Unit of Excellence Institute of Applied Ophthalmobiology (IOBA), University of Valladolid, Valladolid, Spain; 2 Department of Cell Biology, Genetics, Histology and Pharmacology, Faculty of Medicine, University of Valladolid, Valladolid, Spain; 3 Network of Inflammatory Diseases- Networks of Cooperative Research Oriented to Health Results (RICORS), Carlos III National Institute of Health, Madrid, Spain; 4 School of Nursing, University of Valladolid, Valladolid, Spain; 5 Institute of Biomedical Research of Salamanca (IBSAL), University Hospital of Salamanca, University of Salamanca, CSIC, Salamanca, Spain; 6 Institute of Functional Biology and Genomics, CSIC, University of Salamanca, Salamanca, Spain; 7 Department of Ophthalmology, University Clinical Hospital of Valladolid, Valladolid, Spain

**Keywords:** Prognostic markers, Cell death in the nervous system, Retina

## Abstract

Retinal detachment (RD) separates the retina from the retinal epithelium, causing photoreceptor apoptosis and irreversible vision loss. Even with successful surgical reattachment, complete visual recovery is not guaranteed. The *TP53* Arg72Pro polymorphism, implicated in apoptosis, has emerged as a potential predictor of RD outcomes. We investigated the impact of the Arg72Pro polymorphism on retinal neurodegeneration and functional recovery in patients. The underlying mechanisms were analyzed in a humanized *TP53* Arg72Pro RD mouse model. In a cohort of 180 patients, carriers of the Pro allele exhibited decreased apoptotic gene expression and improved visual recovery. Complementary findings in mice revealed that the Pro variant preserved photoreceptor integrity and reduced apoptosis rates following RD. Our findings highlight the potential of this *TP53* polymorphism as a biomarker for RD outcomes and a tool for tailoring therapies. This study underscores the importance of integrating genetic profiling into personalized medicine approaches to improve recovery of RD patients’ visual outcomes.

## Introduction

Retinal detachment (RD) is an ocular emergency defined by separating the neuroretina from the retinal pigment epithelium (RPE). This disruption impacts photoreceptor homeostasis [1]. Rhegmatogenous RD (RRD) is the most prevalent, with an incidence ranging from 10 to 55 per 100,000 individuals annually [1, 2]. This process interrupts the exchange of metabolites between the neuroretina and the choroidal circulation, resulting in ischemia and neurodegenerative changes [1]. Despite advancements in RD repair techniques with high anatomical success, functional outcomes vary significantly [3, 4]. Outcomes are even poorer when RRD is complicated by proliferative vitreoretinopathy (PVR) [5].

Apoptosis plays a critical role in retinal neurodegeneration following retinal detachment (RD) [6, 7]. The death of photoreceptors after RD peaks within three days and is primarily driven by apoptosis, although necrosis and autophagy contribute [6, 7]. Secondary factors such as inflammation and oxidative stress exacerbate apoptosis, creating a neurotoxic environment that further damages the neuroretina [8]. Photoreceptor cell death exhibits significant variability beyond the first seven days, highlighting the possible influence of genetic factors [9, 10]. Identifying shared molecular targets across pathways involving apoptosis and inflammation is crucial to advancing neuroprotective and regenerative therapies. The tumor suppressor protein p53, encoded by the *TP53* gene, plays a central role in regulating apoptosis in response to cellular stress, such as ischemia [11–14]. Among the genetic variants identified in *TP53*, the Arg72Pro (rs1042522) single-nucleotide polymorphism (SNP) is one of the most extensively studied [15]. This variant leads to an arginine-to-proline amino acid substitution, altering the apoptotic function of p53 [16–18]. Located in a proline-rich domain essential for p53’s pro-apoptotic activity, the Arg variant has been linked to increased apoptotic induction and implicated in various pathologies, including neurodegenerative and inflammatory conditions [19–21]. Our group previously demonstrated a significant association between this SNP and PVR, a severe complication of RD surgery [22]. PVR involves abnormal wound healing in an inflammatory environment, challenging retinal reattachment, and visual recovery [5].

This study explores the role of the *TP53* Arg72Pro polymorphism in modulating the molecular mechanisms underlying retinal neurodegeneration following RD and its potential impact on functional outcomes after RD surgery. For this purpose, it integrated clinical data, molecular analysis of human retinal biopsies, and an experimental mouse RD model expressing the human *TP53* Arg72Pro variant.

## Material and methods

### Patients

Consecutive RDD patients from 2021 to 2023 at the Hospital Clínico Universitario de Valladolid (HCUV) and the Institute of Applied Ophthalmobiology at the UVa were included. The primary inclusion criterion was intraocular surgery to repair RD without other ocular conditions. Exclusion criteria included age under 18 years, RRD associated with trauma, traction, or exudative mechanisms; RRD secondary to giant retinal tears exceeding six clock hours, and preoperative PVR classified as higher than grade C [23]. Retinal human samples were obtained by active cutting and aspiration of the retinal flap at the tear site using the vitrectomy cutter (Supplementary video and Fig. S1). All samples were coded and stored in a biobank at −80 °C. Additionally, venous blood samples were collected.

The patient data included demographic characteristics, clinical features, and treatment modality. Patients were treated using pars plana vitrectomy (PPV). Patients who developed visually significant cataracts after RD surgery underwent cataract surgery before any analysis. Best corrected visual acuity (BCVA) was obtained at presentation and 6 months postoperatively and was initially evaluated using the Snellen chart and then converted to the logarithm of the minimum angle of resolution (LogMAR) scale. Visual recovery was the difference between postoperative and preoperative LogMAR visual acuity.

### Swept-Source Optical Coherence Tomography (SS-OCT) and Wide-Field Fundus Autofluorescence (WF-FAF) Imaging

Patients underwent WF-FAF and SS-OCT imaging at baseline and 6 months postoperatively (Optomap P200Tx and DRI OCT Triton). The images were interpreted using the IMAGEnet 6 V image viewer (Topcon) and Optos V2 Vantage software. WF-FAF images were obtained using excitation filters in the green spectrum (about 532 nm). SS-OCT was performed by acquiring a 12-mm × 9-mm three-dimensional-wide scan comprising 256 B-scans, each of which comprised 512 A-scans (512 × 256 A-scans, 512 A-scans for each of 256 B-scans). All imaging procedures followed a standardized acquisition and analysis protocol to ensure cross-site consistency. SS-OCT metrics were derived following predefined segmentation landmarks, with consistent positioning and scan parameters across all centers. Retinal parameters were automatically calculated using the device’s auto-segmentation function (Figs. S2 and S3*)*. Evaluated baseline retinal changes in acute and progressive RRD and RPE-photoreceptor dysregulation using a staging classification system [24]. Only patients with gradable foveal OCT images at presentation were included in this study. The exclusion criteria included patients with any pre-existing macular pathology. Epiretinal membrane (ERM) and cystoid macular edema (CME) were noted on OCT for all patients 6 months post-RD surgery. CME diagnosis is hyporeflective spaces across retinal layers.

### DNA isolation and *TP53* polymorphism analysis

Genomic DNA was isolated from peripheral blood using the Purelink Genomic DNA Mini Kit (Invitrogen) and diluted to 100 ng/μL. Genotyping of the *TP53* Arg72Pro polymorphism was performed by PCR-RFLP. Polymorphism was detected by amplifying genomic DNA with 5‘-TGCCGTTCCCCCTGACATCT-3‘ and 5‘-CTGACCGTGCAAGTCACAGA-3‘ using Dream Taq Hot Start Green PCR Master Mix. A 291-bp fragment containing BstU1-RFLP was amplified. The digests were separated on 2% agarose gel, and the fragments stained with ethidium bromide were analyzed under UV light. To ensure reproducibility, 5% of the samples were re-genotyped, confirming all matched initial genotypes.

### Animals and experimental model of retinal detachment

Humanized *TP53* Arg72Pro knock-in (KI) mice were used for the experimental RD [25]. Experimental RD was induced in 12-week-old *TP53* Arg72Pro mice, per Matsumoto et al. [26]. Male mice were anesthetized with 3% isoflurane (IsoVet, Braun) in an induction chamber using a low-flow system (SomnoSuite, Kent Scientific). Anesthesia was maintained with 1.5% isoflurane through an inhalation mask. RD was performed on the right eye using a surgical microscope. Topically, cyclopentolate 1% (Alcon, Belgium) and phenylephrine 2.5% (Sigma) were administered to induce mydriasis, and oxybuprocaine 0.2% (Benoxi; Unimed Pharma Ltd) as a local anesthetic. The temporal conjunctiva was incised and detached from the sclera. A 30-gauge needle, bevel up, created a sclerotomy 1 mm posterior to the limbus. A scleral tunnel was created, and then a 30-gauge needle was used for paracentesis to reduce intraocular pressure. A 33-gauge needle attached to a NanoFil 10-μL syringe and pump (World Precision Instruments) was inserted into the subretinal space with the bevel down. Then, 4 μL of 1% sodium hyaluronate was injected, detaching the retina from the RPE (Fig. S4*)*. Finally, cyanoacrylate surgical glue (Webglue; Patterson Veterinary) was applied to the scleral wound, and the conjunctiva was reattached to the original position. Excluded were eyes with subretinal hemorrhage or failed detachment analysis. After the surgery, the animals were placed in a warm environment for recovery. Topical ciprofloxacin (Alcon®) lotion was administered every 24 h after surgery. The left eye was used as a control. Animals were sacrificed at 3 and 10 days after RD. Mice were anesthetized by intraperitoneal injection of a mixture (1:4) of xylazine hydrochloride (Rompun, Bayer) and ketamine hydrochloride/chlorbutol (Merial), using 1 ml of the mix per kg of body weight. Finally, the eyes were enucleated for further analysis (Fig. S4).

### Total RNA extraction, reverse transcription, and real-time quantitative PCR

RNA was extracted with the PureLink RNA Mini Kit (Invitrogen). Complementary DNA (cDNA) was synthesized by reverse transcription using a High-Capacity Kit (Applied Biosystems). Relative quantitative real-time polymerase chain reaction (qPCR) was performed using SYBR Green PCR Master Mix (Applied Biosystems) and specific primer sets (Table S1). The qPCR was conducted under the following conditions: 95 °C-10 min, 40 cycles of 95 °C-15 s, 60 °C-1 min, and a final melting curve step. Melting curve analysis was performed to detect primer specificity. *GAPDH* was used as a housekeeping gene. The threshold cycle was determined for each reaction, and gene expression was quantified using the 2^-∆∆Ct^ method [27]. All qPCR reactions were performed in triplicate. The qPCR experiments were conducted in 60 human retinal biopsies and in 24 from the experimental RD-model.

### Enzyme-linked immunosorbent assay

Retinal tissue was lysed using RIPA buffer (Thermo Fisher Scientific). Protein concentrations were determined using the BCA kit (Thermo Fisher Scientific). The protein levels in retinal biopsies (from DR patients and mouse DR model) were measured using commercially quantitative enzyme-linked immunosorbent assay (ELISA) Kits (Table S2). Samples were measured in triplicate, and absorbance was recorded using a SpectraMax M5 spectrophotometer (Molecular Devices) at 450 nm, with wavelength correction set to 620 nm. The protein quantification was performed in 54 retinal biopsies from patients and 24 from the experimental model of RD.

### Histological and immunochemical characterization

Histological and immunochemical characterization was performed in the retinas of mice after RD. Tissue was fixed with 4% paraformaldehyde (Pancreac Quimica) in phosphate-buffered saline (PBS) overnight at 4 °C. Samples were embedded in paraffin (Paraplast Plus, Leica Biosystems) with an automatic tissue processor (ASP300, Leica Microsystems), and five μm sections were obtained. Paraffin-embedded sections were deparaffinized and rehydrated in decreasing ethanol concentrations. Sections were stained with hematoxylin-eosin (Sigma-Aldrich). Sections were processed for immunochemistry by incubation with 0.25% trypsin for 10 min at 37 °C and blocked in PBS with 4% goat serum (Jackson ImmunoResearch Europe) in 0.2% Triton X-100 (Sigma-Aldrich) for 1 h at room temperature (RT). Sections were incubated with primary antibodies diluted in blocking solution: (i) 1:500 anti-GFAP (Z0334, Dako), 1:500 anti-rho (ZRB1-57, Sigma-Aldrich), or 1:100 anti-arrestin (MA5-32156, Invitrogen) for 1 h at RT. Samples were washed with PBS three times and incubated with the (ii) fluorophore-conjugated secondary antibody (Alexa Fluor 568 (red), 1:200, Molecular Probes) in a blocking solution for 1 h at RT. Nuclei were stained with 6-diamidino-2-phenylindole (DAPI, blue fluorescence, 10 µg/mL, Molecular Probes) for 5 min at RT. Fluorescence images were captured using a Leica TCS SP5 DMI-6000B confocal microscope (Leica Microsystems) and analyzed with Leica LAS AF software. Histologic characterization was conducted on three mice for each genotyping group. Quantifications were performed in non-consecutive retinal sections (20X images).

### TdT-mediated dUTP Nick-end Labeling (TUNEL) analysis

The TUNEL kit (11684795910, Roche) detected DNA strand breaks in retinal tissue from the TP53 Arg72Pro mouse model. Retinal sections were fixed and treated as described. After deparaffinization, sections were incubated with 0.25% trypsin in PBS for 10 min at 37 °C and rinsed thrice with PBS. TUNEL reagent was applied for 1 h at RT, and DAPI immunostaining visualized nuclei. The TUNEL assay was conducted on three mice per genotype. Fluorescence images were captured with a Leica TCS SP5 DMI-6000B confocal microscope and analyzed using Leica LAS AF software. TUNEL-positive nuclei were quantified in each animal’s non-consecutive retinal sections (40X).

### Statistical analysis

Quantitative variables are shown as mean ± SD, and qualitative variables as absolute (n) and relative (%) frequencies. When the assumptions of normality or homogeneity of variances were not met, a one-way analysis of variance (ANOVA) or the non-parametric Kruskal-Wallis test to compare quantitative variables between groups (evaluated using the Shapiro-Wilk and Levene tests, respectively) was performed. Post hoc pairwise comparisons were conducted using Bonferroni correction for ANOVA and Dunn’s test for Kruskal-Wallis. Chi-square tests (Fisher’s exact test when expected frequencies were <5) were used to compare qualitative variables between groups. The association between categorical variables was further quantified using odds ratios (OR) and corresponding 95% confidence intervals (CI). Kaplan–Meier curves were constructed using the log-rank test. Ridge coefficients were analyzed to estimate the contribution of variables. A linear regression model was used to evaluate the association between genotype and final BCVA, adjusting for potential confounders. Interaction effects between genotype, foveal status, and surgical timing were assessed using a two-way ANOVA, with visualization of interaction terms provided in the final model. Statistical analyses were performed using SPSS software (IBM Corp., Armonk, NY, USA) and R software. Differences with a *p* < 0.05 were considered statistically significant.

### Study approval

This study involving human subjects was conducted following the Declaration of Helsinki (2008) and received approval from the HCUV Ethics Committee (PI-FIS- 20-1626). The study fully complied with the ethical standards of the World Medical Association, as well as Spanish data protection laws (LO 15/1999) and related regulations (RD 1720/2007). All patients who agreed to participate provided signed written consent. Patients have permitted the publication of their photos. The protocols for all animal experiments were approved by the University of Valladolid’s Institutional Animal Care and Use Committee. They adhered to all relevant international standards and policies, including the European Union Directive on the protection of vertebrates used for experimental and other scientific purposes (2010/63/EU) and according to Spanish law (RD 53/2013). These procedures adhered to the ARVO guidelines for using animals in ophthalmic and vision research. All studies described below were conducted blindly.

## Results

### The *TP53* codon 72 polymorphism is associated with functional outcomes

**Fig. 1:**
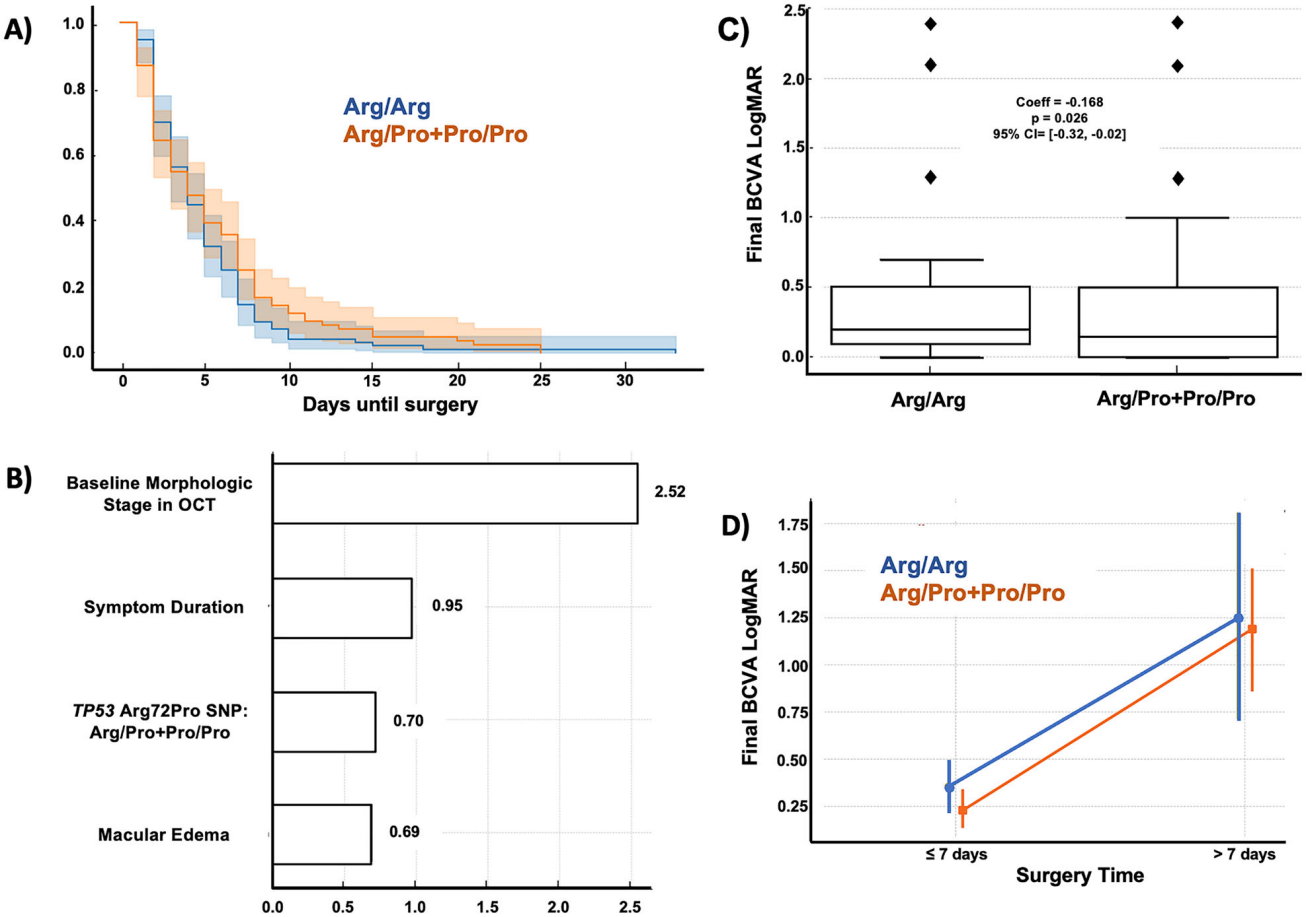
The *TP53* codon 72 polymorphism (Arg72Pro) is associated with functional outcomes after RD. **A** Kaplan–Meier curves illustrate the time until surgery for two genotype groups: Arg/Arg (blue line) and Arg/Pro + Pro/Pro (orange line). The x-axis represents the days until surgery is performed. The Arg/Arg group is more likely to enter surgery earlier than the Arg/Pro + Pro/Pro group. Shaded areas represent the confidence intervals for each curve. **B** Ridge Regression Coefficients for Predicting HRDs. Estimated coefficients from the Ridge regression model predicting the likelihood of HRDs. Each bar represents the coefficient value for the independent variables included in the model: genotype (Arg/Arg vs Arg/Pro + Pro/Pro), baseline morphological stage in OCT, symptom duration (≤10 days vs >10 days), and macular edema (present vs absent). The coefficients indicate each variable’s contribution to the likelihood of exhibiting hyperreflective points. **C** Box plot of final BCVA in LogMAR by genotype (Arg/Arg vs. Arg/Pro + Pro/Pro), based on coefficients from a linear regression model on final BCVA LogMAR. The Arg/Pro + Pro/Pro group showed a slightly better final BCVA, with a genotype coefficient of −0.168 (*p* = 0.026, 95% CI: [−0.32, −0.02]), suggesting a modest visual advantage compared to the Arg/Arg group. Individual points outside the whiskers indicate outliers. This data highlights the potential impact of genotype on visual recovery outcomes. **D** Interaction effects of genotype, foveal status, and surgical timing on final BCVA LogMAR with 95% confidence intervals and *p*-values. The graph illustrates visual acuity (LogMAR) variations across combinations of genotype (Arg/Arg vs. Arg/Pro + Pro/Pro), foveal status (ON vs. OFF), and surgical timing (≤7 days vs. >7 days).

The *TP53* Arg72Pro SNP was analyzed in 180 patients; 96 were Arg/Arg, and 84 were carriers of the Pro variant (Arg/Pro+Pro/Pro). The baseline characteristics showed no statistically significant differences between genotypes (Table 1). BCVA at baseline was better in the Pro carriers, despite this group undergoing surgery later than Arg/Arg subjects (Fig. 1A). During the immediate post-diagnostic period (≤7 days), the Length of Photoreceptor Outer Segments (LPOS) was higher in the Arg/Arg group compared to individuals carrying the Pro allele. Conversely, beyond 7 days following RD diagnosis, LPOS was significantly greater in carriers of the Pro variant (Table 1). A logistic regression model evaluated HRD likelihood, indicating intraretinal inflammatory cells and photoreceptor degeneration (Fig. 1B). The model yielded an overall accuracy of 88.9%. Advanced RD stages (OR = 3.32, 95% CI [1.208, 5.426], *p* = 0.002), be a carrier of the Pro variant (OR = 2.03, 95% CI [0.662, 3.406], *p* = 0.004) and symptom duration (OR = 2.90, 95% CI [1.135, 4.656], *p* = 0.001) were positively associated with HRDs.

**Table 1:** Clinical and surgical OCT parameters according to the *TP53* codon 72 polymorphism (Arg72Pro) at baseline and after a 6-month follow-up.

Gentoype		Arg/Arg				Arg/Pro + Pro/Pro			*p-value*, CI 95%		
Baseline Clinical Characteristics											
*n* (Men/Women)		96 (61/35)				84 (49/35)			0.574		
Age (years) (mean ± SD)		62.06 ± 12.76				60.89 ± 14.12			0.563 [−2.81–5.15]		
Status of the macula (ON/OFF)		21-ON/75-OFF				16-ON/68-OFF			0.787		
Status of the fovea (ON/OFF)		32-ON/64-OFF				29-ON/55-OFF			0.990		
Baseline BCVA (LogMAR), (mean ± SD)		1.17 ± 0.98				1.006 ± 0.845			0.234 [−0.107–0.435]		
Baseline BCVA (LogMAR) by foveal status (ON/OFF), (mean ± SD)		0.38 ± 0.54	1.61 ± 0.78			0.24 ± 0.17	1.32 ± 0.77		0.191 [−0.35–0.07]		0.105 [−0.51–0.04]
Presence of vitreous hemorrhage		6				9			0.280		
Presence of PVR *n* (Grade-B/Grade-C)		11 (5/6)				21 (11/10)			1.000		
Days since symptoms onset (mean ± SD)		12.04 ± 18.38				13.70 ± 16.17			0.763 [−5.90–4.34]		
Time till surgery (mean ± SD)		4.387 ± 4.22				5.65 ± 5.151			0.323 [−2.10–0.71]		
Days since symptoms onset (ON/OFF) (mean ± SD)		5.75 ± 4.09	15.32 ± 20.44			4.22 ± 4.19	16.51 ± 18.32		0.439 [−2.39–5.05]		0.770 [−9.31–6.92]
Time till surgery by foveal status (ON/OFF) (mean ± SD)		2.67 ± 1.42	5.15 ± 4.09			2.73 ± 1.44	6.41 ± 4.83		0.575 [−0.96–1.62]		0.131 [−2.92–0.40]
Status of the lens, (phakic/pseudophakic)		63/33				62/22			0.560		
*Pre-surgical OCT findings*											
Hyperreflective points in the intra/subretinal layer *n* (YES/NO)		76 (22/54)				62 (32/30)			0.011		
Hyperreflective points since symptoms onset, (≤7 days/>7 days)		22 (5/17)				32 (10/22)			0.706		
Baseline Morphologic Stage in OCT *n* (%)	Stage 0	25 (32%)				23 (35.38%)			0.724		
	Stage 1	3 (3.85%)				1 (1.58%)			0.626		
	Stage 2	5 (6.41%)				3 (4.61%)			0.728		
	Stage 3a/3b	20 (25.64%)/12 (15.38%)				18 (27.69%)/9 (13.84%)			0.850/ 0.818		
	Stage 4	7 (8.97%)				5 (7.69%)			1.000		
	Stage 5	6 (7.69%)				6 (9.23%)			0.770		
LPOS* *n* (mean ± SD), (≤7 days/> 7 days)		14 (61.42 ± 26.59)	39 (40.80 ± 24.00)			21 (55.23 ± 45.3)		20 (58.83 ± 23.90)	0.003 [−17.67, 30.05]		0.002 [−30.93, −5.13]
Foveal thickness *n* (mean ± SD)		76 (303.4 ± 73.77)				62 (327.3 ± 79.24)			0.1239 [−54.46–6.668]		
Choroidal thickness *n*, (mean ± SD)		76 (241.5 ± 80.20)				62 (214.0 ± 76.31)			0.0857 [−0.393–58.93]		
Presence of cysts *n* (YES/NO)		60 (45/15)				46 (38/8)			1.000		
Presence of retinal fold *n* (YES/NO)		60 (25/35)				46 (20/26)			1.000		
Clinical Characteristics after 6 months follow-up after surgery											
Final BCVA (LogMAR) (mean ± SD)		0.49 ± 0.71				0.38 ± 0.57			0.2991 [−0.08–0.28]		
Final BCVA (LogMAR) by previous foveal status (ON/OFF) (mean ± SD)		0.10 ± 0.07		0.64 ± 0.76		0.04 ± 0.05		0.48 ± 0.49	0.0003 [0.02–0.09]	0.182 [−0.07–0.39]	
Number of ETDRS letters gained after surgery^a^		34.17 ± 44.17				30.95 ± 33.36			0.5860 [−8.42–14.86]		
Number of letters gained by previous foveal status (ON/OFF)^a^		14.58 ± 27.17		46.93 ± 45.36		10.0 ± 9.05		46.10 ± 28.58	0.390 [−6.01–15.1]	0.906 [−13.1–14.8]	
% (*n*), of FOVEA OFF patients with BCVA 20/40 or higher after surgery		50.88**%** (29)				53.66% (22)			0.6616		
%, (*n*), of FOVEA ON patients with BCVA < 20/20 or lesser		28.12%, (9)				17.24% (5)			0.1163		
Final BCVA (LogMAR) in MACULA OFF RD (<7 d/>7 d) (mean ± SD)		0.44 ± 0.54			0.89 ± 0.95	0.19 ± 0.33		0.78 ± 0.61	0.003 [0.08–0.41]	0.462 [−0.18–0.40]	
Retinal Re-detachment by PVR		4				7			0.3514		
Combined surgery (phaco + PPV)		38				32			0.8789		
*Surgical OCT findings after 6 months follow-up after surgery*											
EZ & ELM Grading**	Grade I	64				55			0.8760		
	Grade II	17				14			1.000		
	Grade III	13				6			0.2246		
	Grade IV	2				9			0.0247		
Central retinal thickness, (*n*) (mean ± SD)		96 (274.4 ± 47.41)				84 (303.3 ± 112.1)			0.0225 [−53.6–−4.12]		
Choroidal thickness, (*n*) (mean ± SD)		96 (183.9 ± 77.69)				84 (172.5 ± 63.45)			0.287 [−9.65–32.45]		
Presence of ERM, % (*n*)		14.58% (14)				32.14% (27)			0.007		
Presence of macular edema, % (*n*)		11.45% (11)				19.04% (16)			0.2091		

*BCVA* best corrected visual acuity (we assigned 2.1 for count fingers vision, 2.4 for hand motions, 2.7 for LP, and 3.0 for no LP, where each increment represents a doubling of the visual angle); *PVR* proliferative vitreoretinopathy, *LPOS* length of photoreceptor outer segments, *PPV* pars plana vitrectomy, *EZ* ellipsoid zone, *ELM* external limiting membrane (EZ & ELM Grading**: for both structures, grade 0 and 1 is defined as a standard and continuous structure, grade 2 as altered but continuous, grade 3 as interrupted and grade 4 as absent), *ERM* epiretinal membrane.

^a^Letters gained= (Baseline LogMAR−Final LogMAR)/0.02. T-*test*, Chi^2^, and Mann–Whitney U for non-parametric tests were used.

Post-RD surgery (Table 1) results revealed that in cases of macula-off RD, final BCVA outcomes were influenced by both the timing of surgery and Arg72Pro polymorphism. Patients undergoing surgery within seven days of symptom onset had better visual outcomes. Those with the Pro allele achieved a slightly improved final BCVA (LogMAR 0.19 ± 0.33) compared to Arg/Arg patients (LogMAR 0.44 ± 0.54). In contrast, those treated after seven days showed poorer visual recovery, with both genotypes presenting worse BCVA (LogMAR 0.89 ± 0.95 in Arg/Arg and 0.78 ± 0.61 in Pro carriers). These findings suggest that the *TP53* Arg72Pro SNP may have a slight advantage in visual outcomes, mainly when intervention occurs early (≤7 days). Although central retinal thickness slightly increased in the Pro patients (*p* = 0.0225), other factors, including choroidal thickness and CME, did not differ significantly between groups. The patients harboring the Pro variant had a higher prevalence of ERM, indicating potential inflammatory complications associated with this genotype. After 6 months, the Pro variant showed a higher risk of retinal re-detachment due to PVR and CME, but differences were not statistically significant. The Pro allele correlated with EZ and ELM grading, especially grade IV (Table 1).

A multivariate regression analysis assessed the effects of genotype, foveal status, and surgery timing on final BCVA. The results indicated that genotype had a slight negative association with final BCVA, suggesting a modest visual advantage in patients harboring the Pro allele (Fig. 1C). The model explained 58% of the variance in final BCVA (R² = 0.58), indicating a good fit and emphasizing the relevance of *TP53* polymorphism, foveal status, and surgical timing in influencing vision outcomes. A multifactorial ANOVA further revealed significant interaction effects among *TP53* codon 72 polymorphism, foveal status, and timing of surgery (Fig. 1D). The best BCVA outcomes were observed in pro-allele carriers, early surgical intervention, and fovea-on status. Conversely, the poorest visual outcomes were found in patients with fovea-off status, the Arg/Arg genotype, and delayed surgery.

### The Arg72-p53 variant triggers apoptotic death during retinal neurodegeneration

**Fig. 2:**
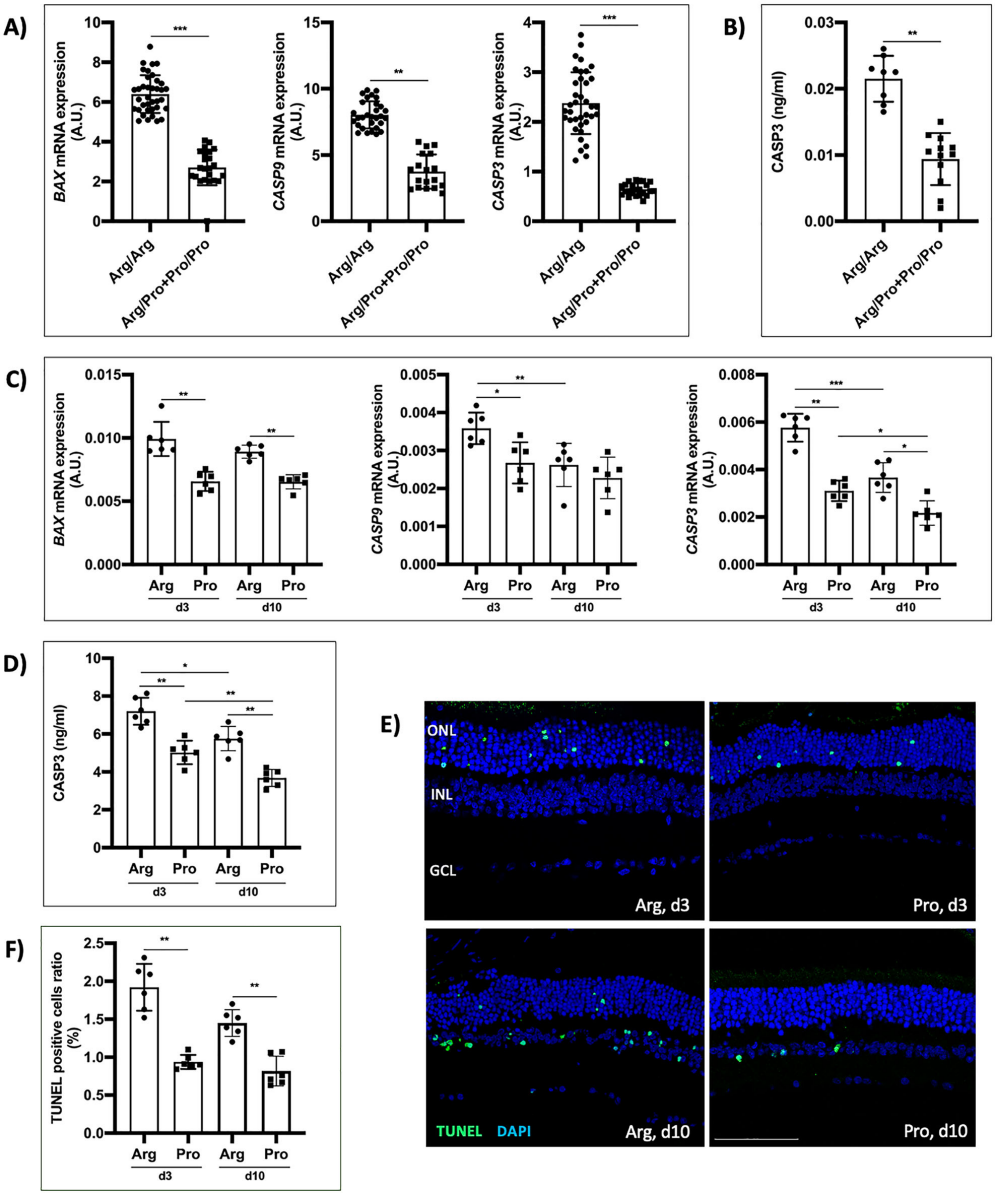
The Arg72-p53 variant is associated with the activation of apoptotic cell death after RD. **A** Relative quantification of *BAX*, *CASP9*, and *CASP3* gene mRNA expression in human retinal samples. **B** Quantification of CASP3 protein in human retinal samples. **C** Relative quantification of *BAX*, *CASP9*, and *CASP3* gene mRNA expression in animal model retinas 3 and 10 days after RD. **D** Quantification of CASP3 protein in animal model retinas at 3 and 10 days after RD. **E** TUNEL assay in animal model retinas 3 and 10 days after RD, scale bar: 75 μm. **F** Quantification of positive nuclei in TUNEL assay in retinas of the animal model, at 3 and 10 days after RD. ONL outer nuclear layer, INL inner nuclear layer, GCL ganglion cell layer, *P < 0.05, **P < 0.01, ***P < 0.001, AU arbitrary units. Bars represent mean values and their respective standard deviation.

Once demonstrated that the *TP53* Arg72Pro SNP dictates visual improvement after RD, and considering the key role of retinal neurodegeneration in this process, we next evaluated the impact of the SNP on the activation of apoptotic cell death. The results showed that the expression of *BAX*, *CASP9*, and *CASP3* genes in retinal tissue from homozygous Arg patients was higher than in patients carrying the Pro variant (Fig. 2A). The CASP3 protein level was also higher in retinal biopsies from the homozygous Arg allele patients (Fig. 2B).

Accordingly, it was observed that the expression of *BAX, CASP9*, and *CASP3* genes in retina tissue at day 3 was higher in the mice harboring the Arg allele. On day 10, *BAX* and *CASP3* gene expression were higher in 72Arg-p53 mice than in 72Pro-p53 mice. The expression of *CASP9* and *CASP3* genes was higher in the 72Arg-p53 mice on day 3 than on day 10. The *CASP3* gene expression was higher in 72Arg-p53 mice on day 3 than on day 10 (Fig. 2C). In the case of the CASP3 protein, its expression was also higher in 72Arg-p53 mice, both at day 3 and 10 (Fig. 2D). The TUNEL staining revealed more apoptotic cell death at days 3 and 10 in the mice carrying the Arg allele (Fig. 2E, F).

### The inflammatory and Müller cell’s response is associated with the Pro72-p53 allele

**Fig. 3:**
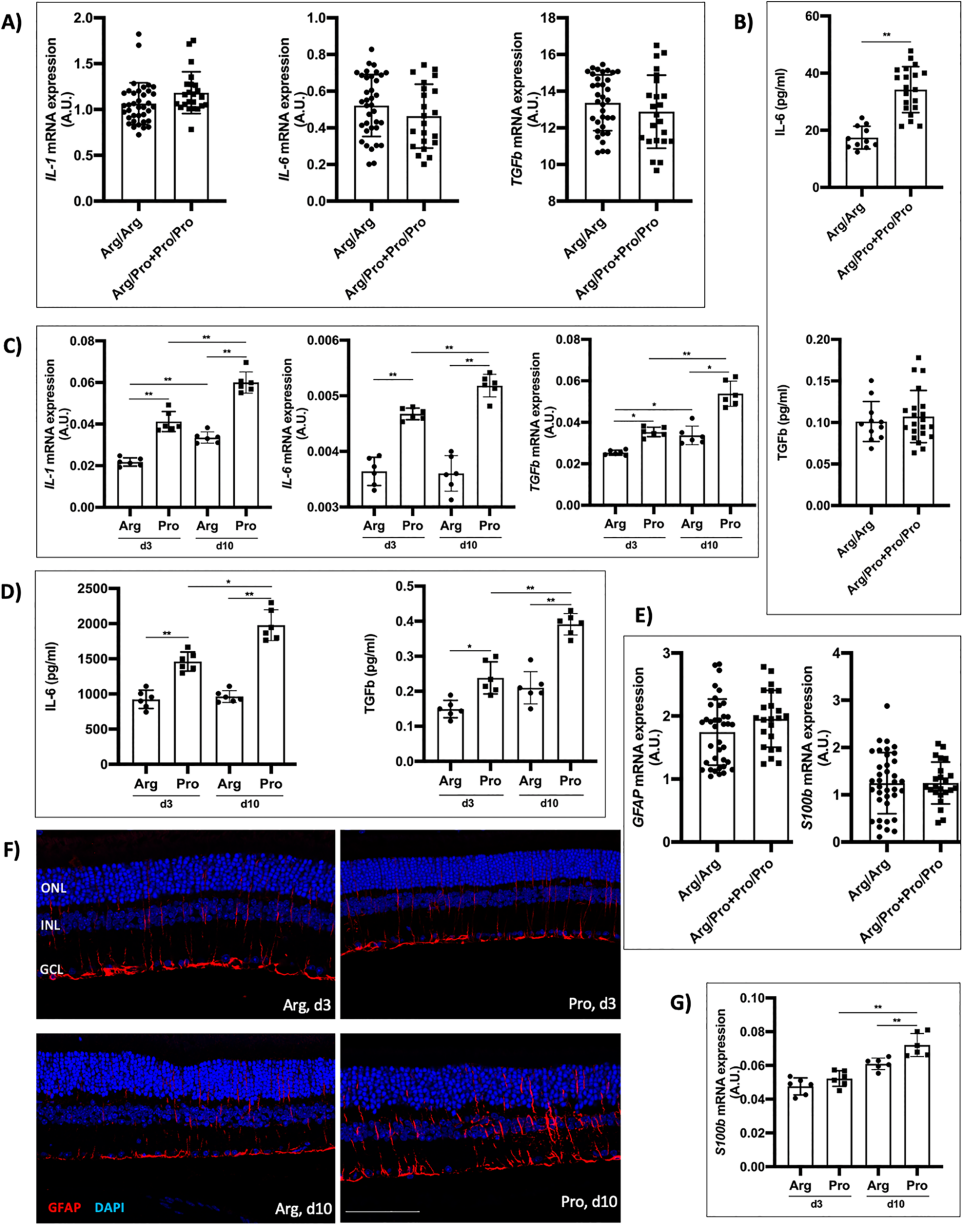
The Pro72-p53 variant is related to increased inflammation after retinal RD. **A** Relative quantification of mRNA expression of *IL-1*, *IL-6*, and *TGFb* genes in human retinal samples. **B** Quantification of IL-6 and TGFb proteins from human retinal samples. **C** Relative quantification of mRNA expression of *IL-1*, *IL-6*, and *TGFb* genes in animal model retinas at 3 and 10 days after RD. **D** Quantifying IL-6 and TGFb proteins in animal model retinas 3 and 10 days after RD. **E** Relative quantification of mRNA expression of *GFAP* and *C100b* genes in human retinal samples. **F** Immunoreactivity of GFAP in animal model retinas at 3 and 10 days after RD, scale bar: 75 μm. **G** Relative quantification of *C100b* gene mRNA expression in animal model retinas 3 and 10 days after RD. ONL outer nuclear layer, INL inner nuclear layer, GCL ganglion cell layer, *P < 0.05, **P < 0.01, ***P < 0.001, AU arbitrary units. Bars represent mean values and their respective standard deviation.

Considering the essential role of inflammation post-RD, we also examined the impact of the *TP53* polymorphism. The expression of inflammation-related genes in human retinal biopsies yielded no statistically significant differences (Fig. 3A). However, the IL-6 protein levels were higher in patients with the Pro allele (Fig. 3B). In the mouse model, the results showed that the expression of *IL-1*, *IL-6*, and *TGFb* genes, at days 3 and 10, was higher in the mice harboring the Pro allele than in those with the Arg variant. Their expression was higher in the 72Pro-p53 mice on day 10 than on day 3 (Fig. 3C). The levels of the IL-6 and TGFb proteins were higher in the 72Pro-p53 mice than in the 72Arg-p53 mice on days 3 and 10. In the 72Pro-p53 mice, the expression was higher at day 10 than at day 3 (Fig. 3D).

Inflammation constitutes a crucial cellular process during retinal neurodegeneration, and glial cells play a central role. Therefore, the response of retinal Müller cells after RD was analyzed. Glial fibrillary acidic protein (GFAP) is expressed in Müller cells in response to neurodegeneration [28]. *GFAP* expression in human retinal biopsies showed no statistically significant differences (Fig. 3E). S100B is a calcium-binding protein mainly concentrated in astrocytes and other glial cell types, such as retinal Müller cells, and is considered a biomarker of neuronal distress [29]. The *S100b* expression did not show differences in human retinal biopsies (Fig. 3E). The GFAP immunoexpression was higher in the mice carrying the Pro allele than in those with the Arg variant. It was observed that the prolongations of the glial cells invaded the INL and ONL, and it was more pronounced in the 72Pro-p53 mice and even more on day 10 than on day 3 after RD. In the 72Pro-p53 mice, at day 10, the GFAP was distributed throughout the GCL, IPL, INL, and the outermost ONL region (Fig. 3F). The *GFAP* expression was also higher in 72Pro-p53 mice than in 72Arg-p53 mice, with the highest levels at day 10. Finally, the highest expression of the *S100b* gene was observed at day 10 in the mice harboring the 72Pro variant (Fig. 3G).

### The *TP53* Arg72Pro variant modifies the NF-kB and cFOS expression

**Fig. 4:**
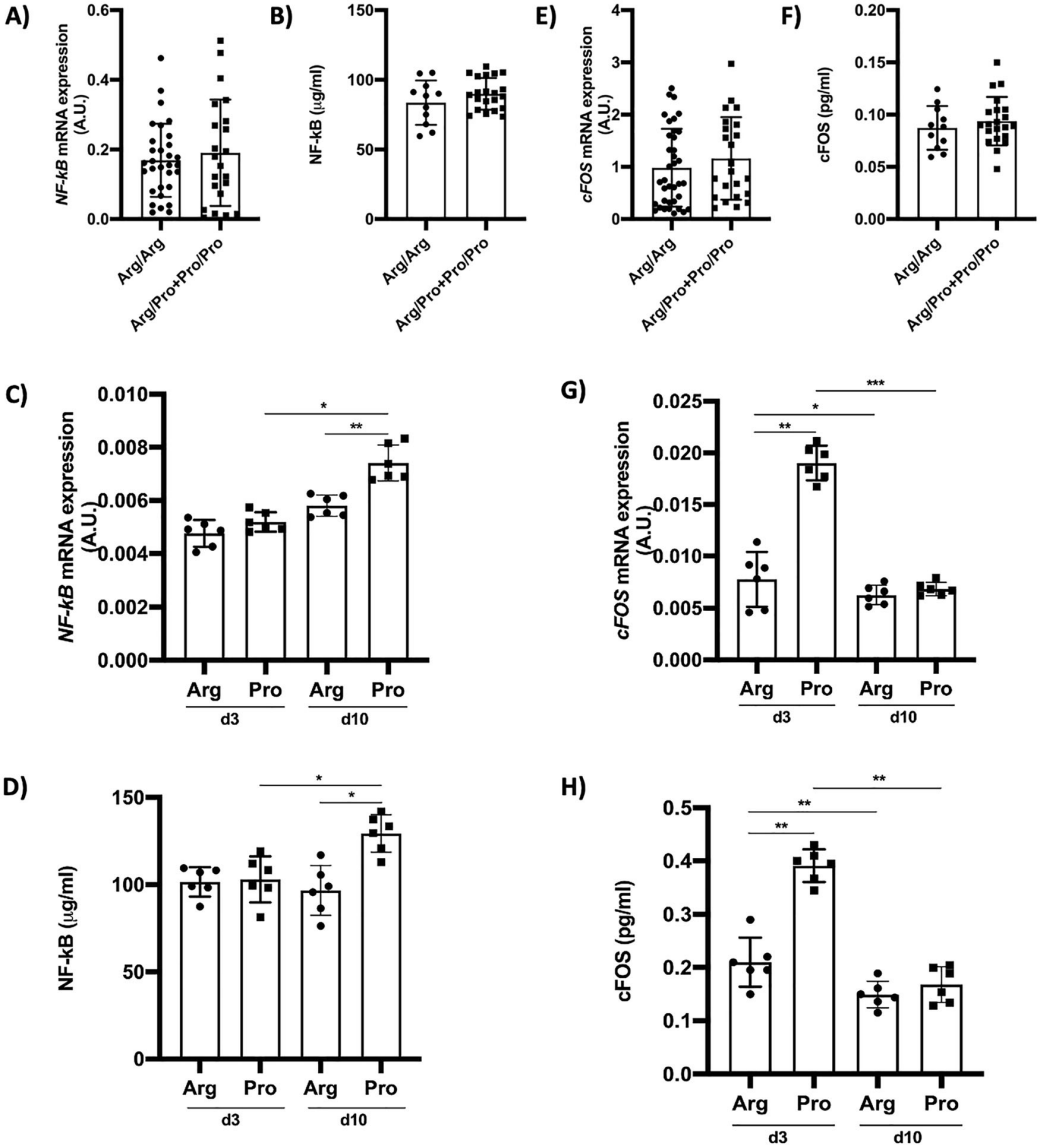
NF-kB and cFos expression could be determined by *TP53* Arg72Pro polymorphism after RD. **A** Relative quantification of mRNA expression of *NF-kB* gene in human retinal samples. **B** Quantification of NF-kB protein in human retinal samples. **C** Relative quantification of mRNA expression of *NF-kB* gen in animal model retinas at 3 and 10 days after RD. **D** Quantifying NF-kB protein in animal model retinas 3 and 10 days after RD. **E** Relative quantification of mRNA expression of *cFos* gene in human retinal samples. **F** Quantification of cFOS protein in human retinal samples. **G** Relative quantification of mRNA expression of *cFOS* gen in animal model retinas at 3 and 10 days after RD. **H** Quantifying cFOS protein in animal model retinas 3 and 10 days after RD. *P < 0.05, **P < 0.01, ***P < 0.001. AU arbitrary units. Bars represent mean values and their respective standard deviation.

The results showed no differences in the NF-kB expression in retinal biopsies from patients (Fig. 4A, B). NF-kB is a stress-inducible transcription factor that plays a central role in regulating immunity and inflammation response [30]. The *NF-kB* expression was higher, at day 10, in 72Pro-p53 mice than in the 72Arg-p53 mice. The expression was higher at day 10 than at day 3 (Fig. 4C). Similar results were obtained for protein levels (Fig. 4D). The immediate early gene *cFOS* has long been known as a biomarker of neural activity [31]. The *cFOS* expression in the human retina did not yield differences (Fig. 4E, F). At day 3, *cFOS* expression was higher in the 72Pro-p53 mice. In the 72Pro-p53 mice, its expression was higher at day 3 than at day 10 (Fig. 4G). These results were similar for the cFOS protein (Fig. 4H).

### The *TP53* Arg72Pro SNP conditions autophagy modulation

**Fig. 5:**
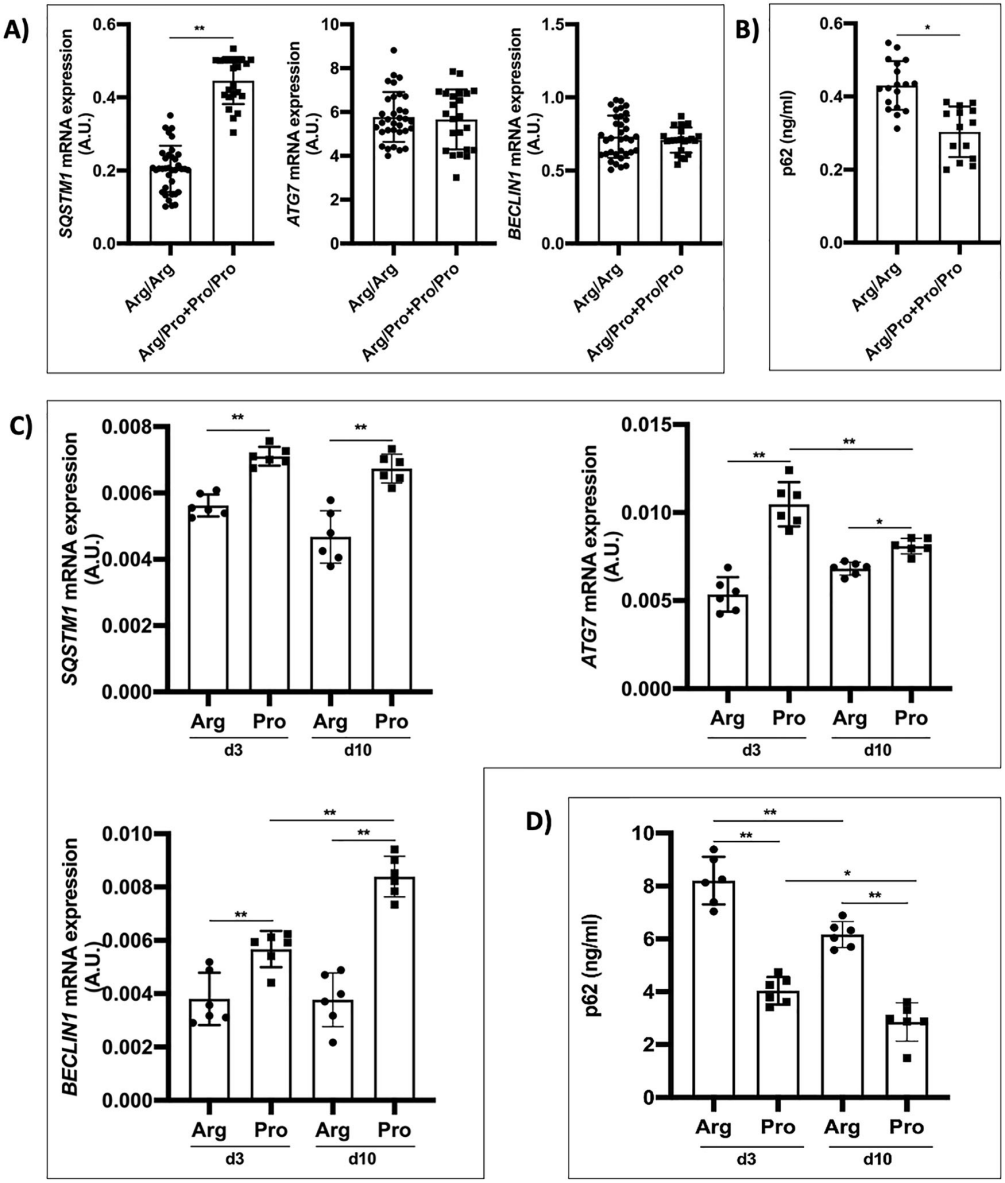
The *TP53* Arg72Pro polymorphism is involved in the autophagy modulation after RD. **A** Relative quantification of mRNA expression of *SQSTM1*, *ATG7*, and *BECLIN1* genes in human retinal samples. **B** Quantification of p62 protein in human retinal samples. **C** Relative quantification of mRNA expression of *SQSTM1*, *ATG7*, and *BECLIN1* genes in animal model retinas at 3 and 10 days after RD. **D** Quantifying p62 protein in animal model retinas 3 and 10 days after RD. *P < 0.05, **P < 0.01, ***P < 0.001. AU arbitrary units. Bars represent mean values and their respective standard deviation.

Considering the role of autophagy during retinal neurodegeneration and its modulation after inflammatory processes, we studied if TP53 SNP modulated its activation. In patients, an increased expression of the *SQSMT1* was observed in carriers of the Pro allele (Fig. 5A). The p62 protein levels were higher in Arg/Arg patients than in the Pro variant carriers(Fig. 5B). The expression of *SQSTM1*, *ATG7*, and *BECLIN1* genes on days 3 and 10 was higher in 72Pro-p53 mice (Fig. 5C). The p62 protein levels, at days 3 and 10, were higher in the mice harboring the Arg allele. The p62 protein levels were higher at day 3 than at day 10(Fig. 5D).

### Influence of *TP53* Arg72Pro polymorphism in the retina morphology after RD

**Fig. 6:**
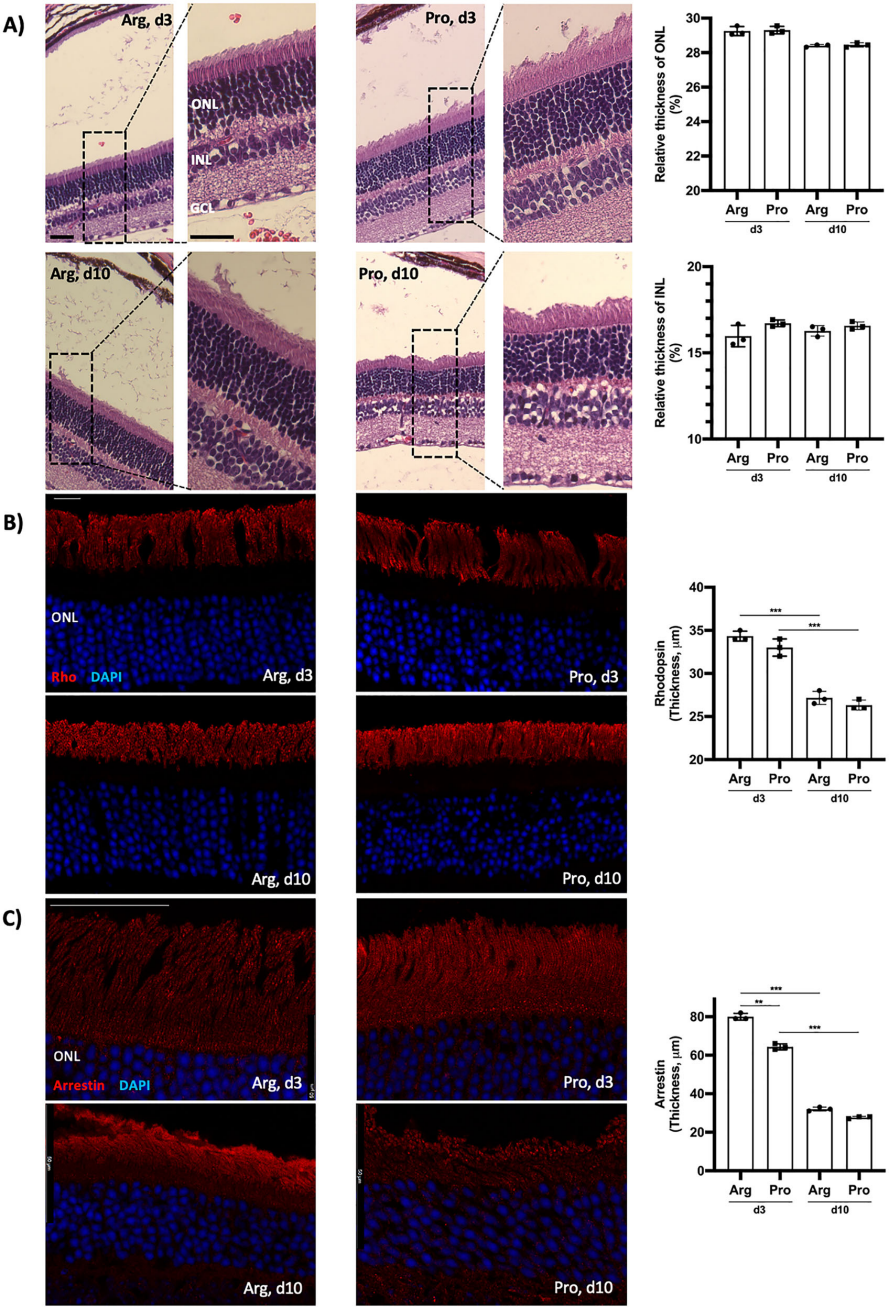
Influence of *TP53* Arg72Pro polymorphism in the photoreceptor morphology after RD. **A** Hematoxylin-Eosin (H-E) stained section from animal model retinas at 3 and 10 days after RD, scale bar: 25 μm. **B** Immunoreactivity of rhodopsin (Rho) from animal model retinas at 3 and 10 days after RD, scale bar: 10 μm. **C** Immunoreactivity of cone arrestin from animal model retinas 3 and 10 days after RD, scale bar: 50 μm. *P < 0.05, **P < 0.01, ***P < 0.001, ONL outer nuclear layer, INL inner nuclear layer, GCL ganglion cell layer.

Changes in retinal morphology associated with the *TP53* Arg72Pro polymorphism after RD were also analyzed. The results showed no differences in the retinal structure. The INL and ONL thicknesses were similar for both genotypes after RD (Fig. 6A). To evaluate whether *TP53* polymorphism affects the morphology of photoreceptors, retinas were immunolabeled with antibodies against cone-arrestin and rho. Rho is inserted into newly forming membrane discs at the base of the outer segments of the rod [32]. No differences were observed in the rho immunoexpression. The rod degeneration was evident between days 3 and 10 after RD (Fig. 6B). Arrestin-4 was used to study cones [33]. On day 3, the arrestin immunoreactivity thickness was higher in the mice carrying the Arg allele than in those with the Pro variant. The cone structure was less well preserved in Arg-mice, with an elongated and edematous appearance (Fig. 6C). In contrast, cone photoreceptors in 72Pro-p53 mice exhibited a more regular morphology. Additionally, cone degeneration was markedly pronounced on day 10 post-RD in both genotypes (Fig. 6C).

### Molecular analysis according to the baseline clinical characteristics

**Fig. 7:**
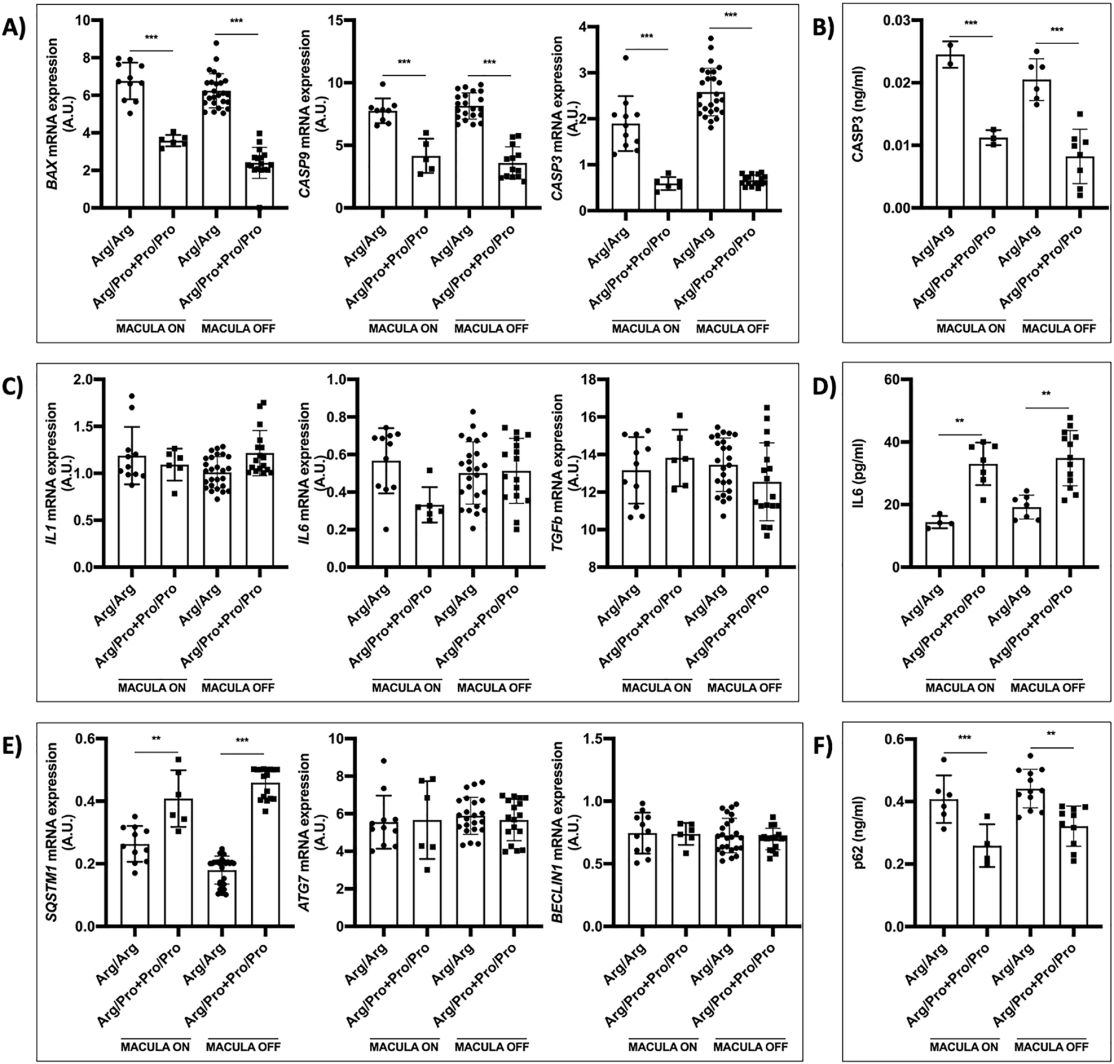
Analysis of apoptosis, inflammation, and autophagy according to the *TP53* Arg72Pro polymorphism and the macula status after RD. **A** Relative quantification of *BAX*, *CASP9*, and *CASP3* gene mRNA expression in human retinal samples. **B** Quantification of CASP3 protein in human retinal samples. **C** Relative quantification of mRNA expression of *IL-1*, *IL-6*, and *TGFb* genes in human retinal samples. **D** Quantification of IL-6 protein in human retinal samples. **E** Relative quantification of mRNA expression of *SQSTM1*, *ATG7*, and *BECLIN1* genes in human retinal samples. **F** Quantification of p62 protein in human retinal samples. *P < 0.05, **P < 0.01, ***P < 0.001. AU arbitrary units. Bars represent mean values and their respective standard deviation.

Molecular analysis was also performed according to the status of the macula and fovea. *BAX*, *CASP9*, and *CASP3* expression was higher in Arg/Arg patients than in carriers of the Pro variant (Fig. 7A). In macula-on and macula-off patients, the levels of the Casp3 protein were also higher in retinal biopsies of the Arg/Arg patients (Fig. 7B). The expression of inflammatory-related genes showed no differences (Fig. 7C). The IL-6 levels were higher in retinal tissue from patients with the Pro allele, in macula-on and macula-off patients (Fig. 7D). About autophagy, increased expression of the *SQSTM1* gene was observed in patients with macula-on and macula-off carrying the Pro allele (Fig. 7E). The p62 protein level was lower in macula-on and macula-off patients harboring the Pro variant (Fig. 7F). The study was also performed according to the fovea status, and the results were very similar to those obtained according to macular status (Fig. S5).

## Discussion

Although anatomical RD repair is often successful, many patients experience significant vision loss [3, 4]. The initial neurodegeneration following RD arises from ischemia caused by the detachment of the neuroretina from the RPE [34–39]. Photoreceptors rely on the RPE for metabolic support, and prolonged ischemia intensifies neurodegeneration, apoptosis, and inflammatory activation [34–39], contributing to the variability in functional outcomes [40, 41]. Genetic factors may influence these processes, modulating the variability in visual recovery [42]. Among these, p53 plays a crucial role as a regulator of apoptosis and neuroinflammation [43, 44]. As a cellular sensor for stress stimuli, including ischemia, p53 has been implicated in assessing the degree of neurodegeneration [12]. Our findings suggest that the *TP53 Arg72Pro* polymorphism dictates RD outcomes by modulating apoptotic activation. The analysis of clinical and OCT evaluations identified the Pro variant as a significant predictor of HRDs, linked to photoreceptor degeneration and inflammation cells. Results also demonstrated that the best BCVA outcomes occurred in patients with the Pro variant who had early surgery and fovea-on status. These results emphasize that *TP53* Arg72Pro polymorphism and timely surgical intervention are critical determinants of visual recovery after RD. This study reinforces the idea that genetic variations and apoptosis-inflammation-driven mechanisms are crucial in the pathophysiology of RD and its postoperative outcomes, highlighting the necessity to integrate genetic information to enhance patient outcomes.

In response to ischemia, stabilized p53 partially translocates to the mitochondria, initiating pro-apoptotic responses [45, 46]. The *TP53-*Arg variant has been associated with an enhanced ability to induce apoptosis in neurons, increasing neuronal susceptibility to ischemia [19]. These findings are consistent with current results, which identified increased retinal cell apoptosis following RD in Arg/Arg individuals. In the mouse model, apoptosis was notably higher within the first three days after RD, especially in mice harboring the Arg allele. In RD patients, LPOS levels were higher in Arg/Arg individuals during the immediate postoperative period. This pattern was also observed in the mouse model, where 72Arg-p53 mice exhibited cone elongation (swelling-like appearance) during the first three days post-RD compared to 72Pro-p53 mice. These results suggest that the *TP53* Arg72Pro genetic variant may partially mediate the initial photoreceptor response to RD.

The clinical follow-up of the included patients showed that Arg/Arg subjects who underwent surgery within the first 7 days experienced worse outcomes than Pro variant carriers. This may be linked to increased retinal cell apoptosis associated with the Arg variant. Previous studies have demonstrated that the Arg72Pro polymorphism influences ischemic tolerance in neurons after stroke [19–21], suggesting that this SNP could also impact the ischemic tolerance of retinal cells after RD and thereby affect the clinical progression of patients, particularly during the initial days post-RD. Current findings further revealed elevated cFos expression on day 3 post-RD in Pro-mice, a variant associated with lower apoptosis activation. As cFos is a known biomarker of neural activity [31], the Pro-variant may enhance ischemic tolerance in the early days after RD. This increased tolerance could support higher retinal neuron activity and improve clinical outcomes in patients with the Pro allele.

Detachment of the neuroretina activates a pro-inflammatory response and exacerbates retinal cell death [36–39]. Our findings show that the inflammatory response after RD is influenced by the Arg72Pro SNP. Specifically, the Pro allele was associated with increased expression of pro-inflammatory cytokines, which peaked at day 10. Consistent with these results, Pro allele carriers showed a higher incidence of OCT-detected complications, such as ERM and CME, driven by inflammatory processes [47, 48]. Moreover, the Pro variant predicted HRDs composed of degenerative and intraretinal inflammatory cells, underscoring its role in modulating inflammation [49, 50]. The NF-κB factor is a key regulator of the inflammatory response and interacts with p53 in various stress-related processes [30, 51]. The Arg72Pro SNP modulates this interaction, with the Pro variant linked to an enhanced inflammatory response [52]. This is consistent with reports associating the Pro variant with diseases characterized by inflammation-driven pathophysiology [53, 54]. Our results demonstrated higher NF-κB levels in 72Pro-p53 mice at day 10, corresponding to the increased inflammatory response. These findings align with our previous studies, which identified the Pro variant as a risk factor for PVR, a highly inflammatory condition, and a leading cause of RD surgical failure [5]. Lastly, the response of retinal Müller cells to RD is well-documented [55]. This study found that this glial response may also depend on the *TP53* Arg72Pro variant; the Pro-variant showed increased glial activity. These findings highlight the dual role of the *TP53* Arg72Pro polymorphism in modulating apoptotic and inflammatory pathways following RD, offering insights into genetic influences on clinical outcomes and potential therapeutic targets.

Increased inflammation disrupts homeostasis, and autophagy compensates to mitigate the response [56, 57]. P53 has a dual role in autophagy regulation, acting as an inducer and a repressor [58, 59]. Our study revealed that the Pro-variant was associated with increased autophagy activation after RD. This finding aligns with the observed heightened inflammatory response in Pro carriers, suggesting that autophagy may be a compensatory mechanism to counteract the inflammation associated with the Pro variant. P62 is a selective adaptor protein that facilitates autophagosome formation and subsequent degradation during autophagy. Therefore, p62 levels are a marker for monitoring autophagy [60]. Analysis after RD showed reduced p62 protein levels, with the highest autophagy activation observed for 10 days in pro-allele carriers. This coincided with the peak inflammatory response in the 72Pro-p53 variant, reinforcing the hypothesis that autophagy is activated as a response to inflammation. The activation of autophagy following RD has previously been associated with retinal cell survival [61]. Our findings support this hypothesis, as reduced retinal cell apoptosis was observed in carriers of the Pro allele. This suggests that increased autophagy may contribute to retinal cell protection in the presence of the Pro variant despite the heightened inflammatory response. Collectively, these results highlight the dual role of the Pro allele in modulating both inflammation and autophagy, providing insights into the complex mechanisms underlying retinal cell survival and neurodegeneration after RD.

This study highlights several notable strengths that underscore the significance and reliability of its findings. The main strength is its translational approach, combining patient clinical data, molecular analyses of human retinal biopsies, and an experimental mouse model. This integration bridges the gap between molecular mechanisms and clinical outcomes, enhancing the study’s validity and practical relevance. Incorporating clinical biomarkers alongside the *TP53* Arg72Pro polymorphism also has significant implications for personalized medicine, supporting preoperative patient stratification and individualized treatment planning. However, the study has limitations. One limitation is the variability inherent in the molecular analyses of human retinal biopsies, as sample heterogeneity may affect the precision and reproducibility of biomarker-related findings. Additionally, the sample size warrants attention. Although our cohort provided valuable insights, certain comparisons did not achieve statistical significance. Expanding the sample size in future studies could improve statistical power and clarify these relationships.

This research emphasizes the different effects of the *TP53* Arg72Pro SNP on retinal neurodegeneration following RD. The Arg variant is strongly associated with increased retinal cell apoptosis, particularly within the first three days post-RD. In contrast, the Pro allele is linked to heightened inflammatory and autophagy responses, becoming most evident around day 10. These findings provide valuable insights into observed clinical outcomes: Arg/Arg patients operated on within seven days of RD showed worse functional outcomes than Pro variant carriers, while Pro allele carriers operated on after seven days had the poorest outcomes overall. These results underscore the value of incorporating genetic insights into treatment planning to enhance early intervention strategies and achieve better patient outcomes. Patient analysis stratified by the *TP53* Arg72Pro SNP and baseline clinical characteristics yielded results consistent with these observations, showing no significant differences based on macular or foveal involvement at baseline. However, the observed postoperative outcomes highlight the potential influence of the *TP53* Arg72Pro polymorphism on visual recovery, which may not be fully explained by baseline clinical characteristics alone.

In conclusion, this study underscores the critical role of the *TP53* Arg72Pro SNP in modulating retinal neurodegeneration following RD. These processes significantly influence the functional and clinical recovery of RD patients. Thus, the *TP53* Arg72Pro polymorphism could be a molecular biomarker to predict functional prognosis in RD patients, guiding personalized treatment strategies.

## Supplementary information

Supplementary material

Supplementary Video 1

## Data Availability

All data needed to evaluate the conclusions in the paper are present in the paper and/or the Supplementary Materials. Additional data related to this paper may be requested from the authors.
