# Supplementary material for: The TP53 Arg72Pro polymorphism predicts visual and neurodegenerative outcomes in retinal detachment
Source: Cell Death Dis. 2025 May 26;16(1):415. doi: 10.1038/s41419-025-07739-1 (PMC12106684; doi:10.1038/s41419-025-07739-1)
Supplement: Supplementary file 1 — Supplementary material [file 41419_2025_7739_MOESM1_ESM.docx]

**Supplementary material**

**Supplementary video. Collection of human retinal biopsies.** Retinal samples were obtained by active cutting and aspiration of the retinal flap at the tear site using the vitrectomy cutter

**
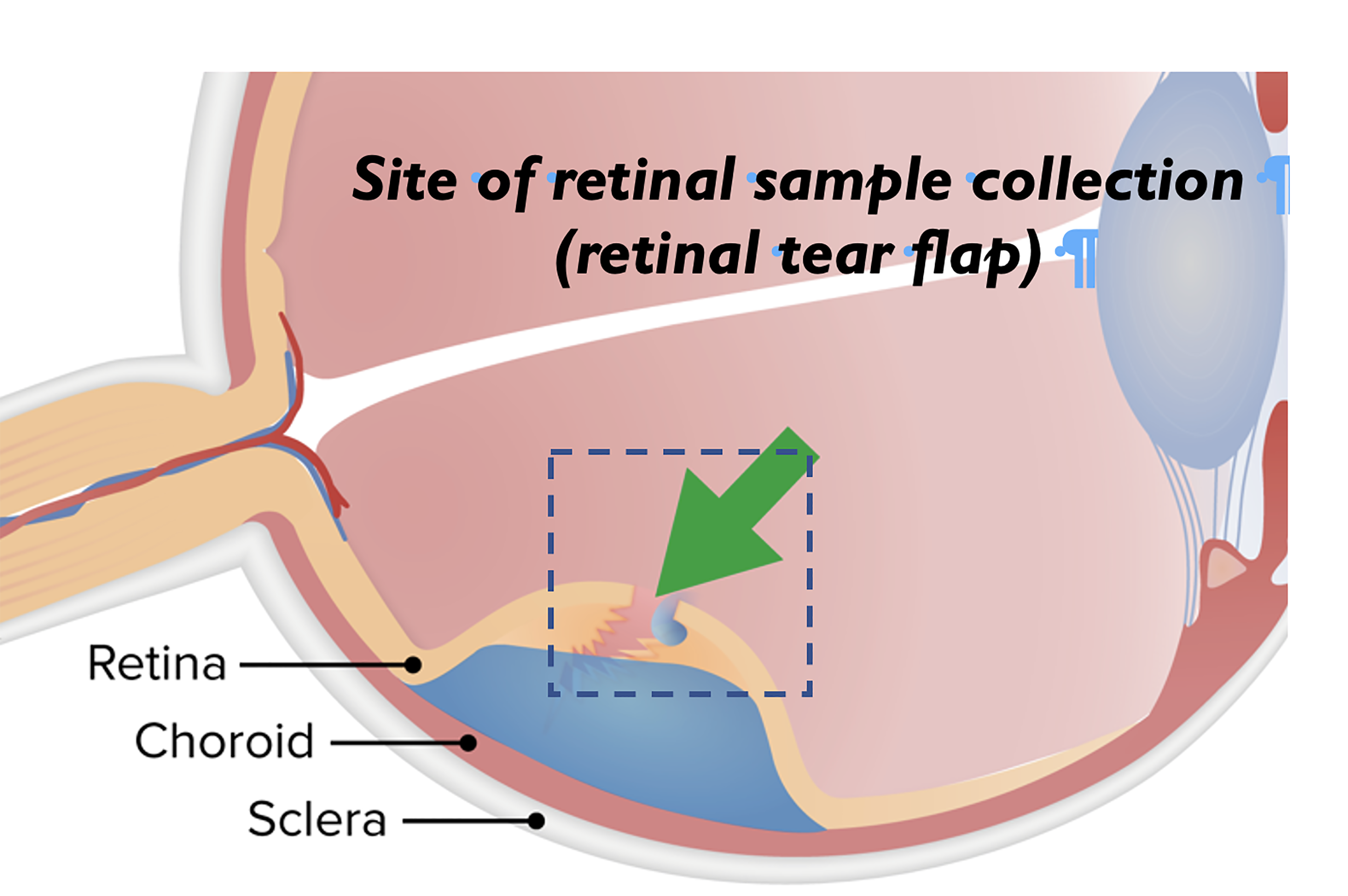
**

**Fig S1. Diagram of the human eye illustrating a retinal detachment caused by a retinal break**. It shows the separation of the retina from the choroid and sclera. The green arrow marks where the retinal sample is collected (retinal tear flap) using a vitrectomy probe.

**
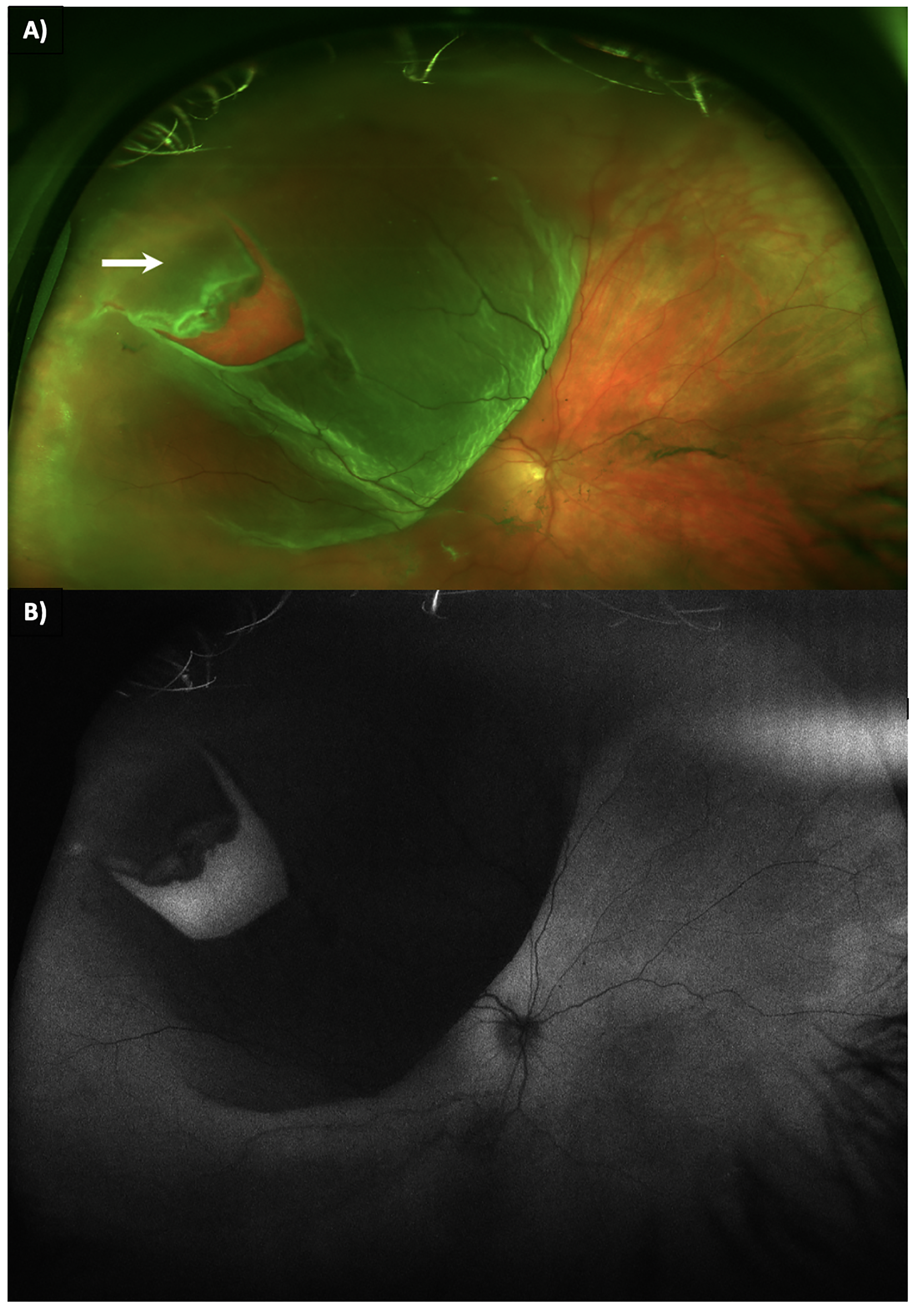
**

**Fig S2. Rhegmatogenous Retinal Detachment (RRD).** (A) Widefield fundus photograph depicting bullous macula-off RRD with a visible retinal break, marked by a white arrow. (B) Widefield fundus autofluorescence image highlighting a hyperfluorescent area corresponding to subretinal fluid in the same patient with macula-off RRD.

**
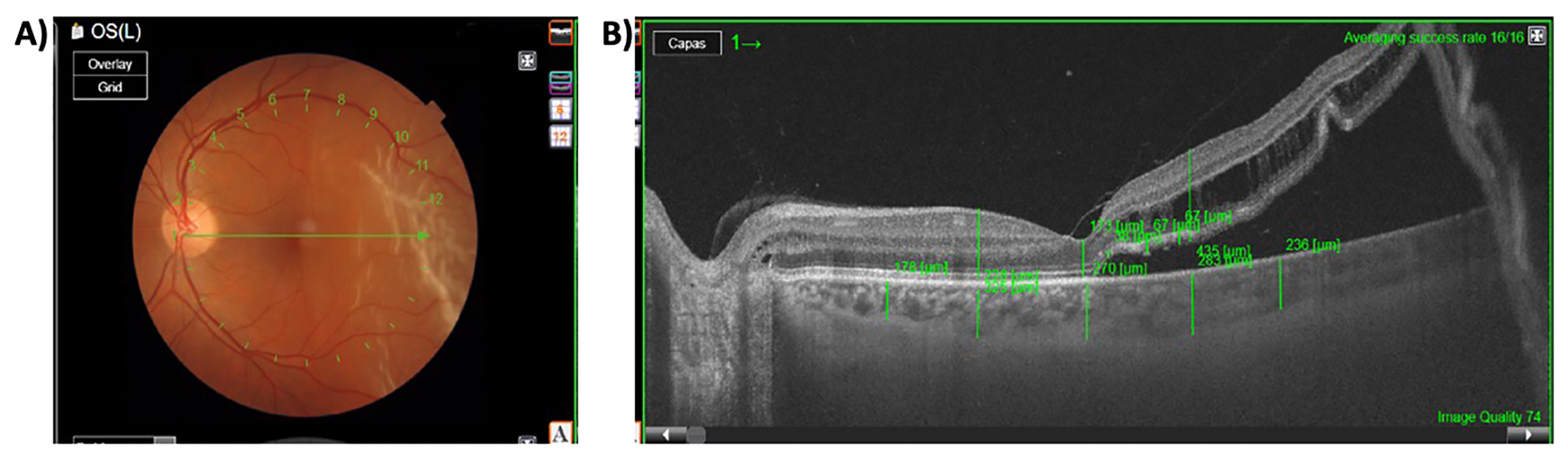
**

**Fig S3. Swept-source Optical Coherence Tomography (SS-OCT) images. Representative multimodal imaging protocol, qualitative and quantitative SS-OCT measurements.** (A) Fundus image of a patient with macula-off rhegmatogenous retinal detachment (RRD), illustrating the detached retina and associated structural changes. (B) Cross-sectional SS-OCT image showing detailed retinal and choroidal architecture. *Subfoveal Choroidal Thickness:* Defined as the distance between the outer border of the retinal pigment epithelium–Bruch’s membrane complex and the chorioscleral border directly under the fovea. Five independent measurements were performed to enhance measurement accuracy and ensure reproducibility. *Central Retinal Thickness:* Measured in non-detached areas as the distance between the outer surface of the neurosensory retina and the retinal pigment epithelium. In detached regions, measurements also included the outer segment of photoreceptors. For consistency, three central measurements were recorded in all cases. *Photoreceptor Outer Segment Length:* The thickness of the total photoreceptor layer and the specific outer segment layer was measured separately. Calipers were used to ensure precision, and all measurements were annotated for clarity and consistency.


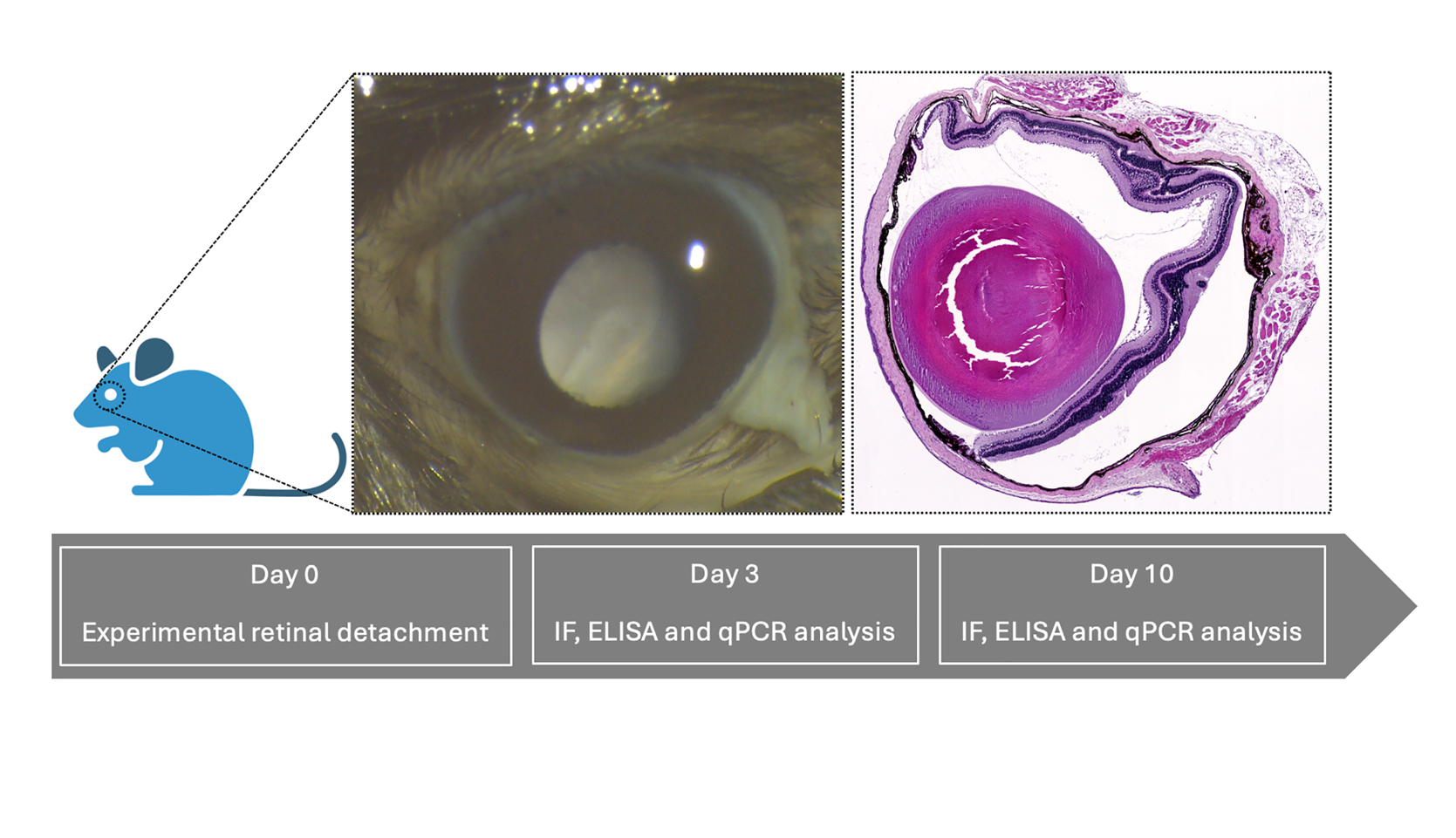


**Fig S4. Methodological design for evaluating the experimental retinal detachment in the murine model.** Fundus and histologic image after experimental RD. IF: immunofluorescence, ELISA: Enzyme-Linked Immunosorbent Assay, and qPCR: real-time quantitative comparative PCR.


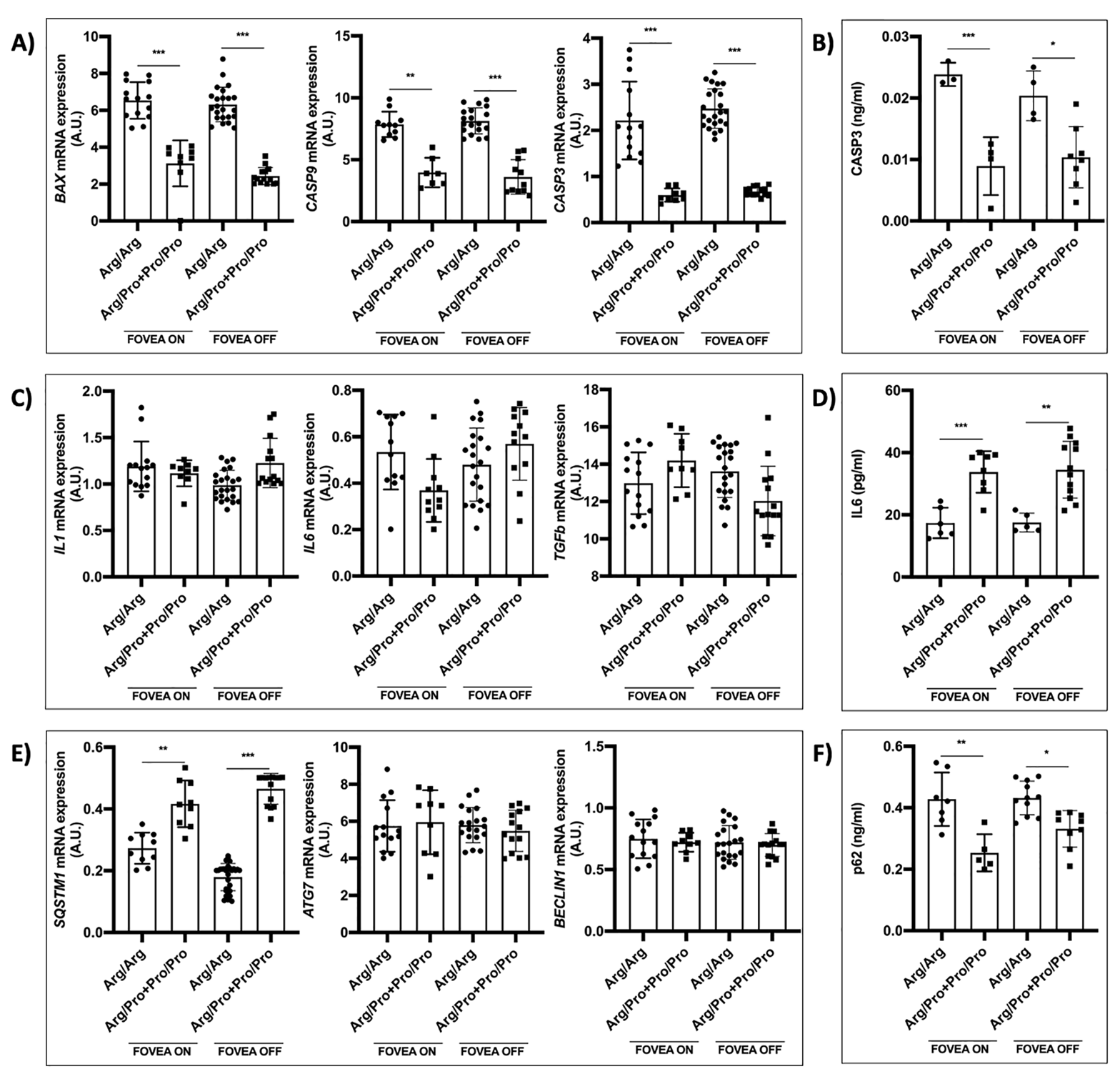


**Fig S5. Analysis of apoptosis, inflammation, and autophagy according to the *TP53* Arg72Pro polymorphism and the macula status after RD.** (A) Relative quantification of *BAX*, *CASP9*, and *CASP3* gene mRNA expression in human retinal samples. (B) Quantification of CASP3 protein in human retinal samples. (C) Relative quantification of mRNA expression of *IL-1*, *IL-6*, and *TGFβ* genes in human retinal samples. (D) Quantification of IL-6 protein in human retinal samples. (E) Relative quantification of mRNA expression of *SQSTM1*, *ATG7*, and *BECLIN1* genes in human retinal samples. (F) Quantification of p62 protein in human retinal samples. *:P < 0.05, **:P<0.01, ***:P<0.001. AU: arbitrary units. Bars represent mean values and their respective standard deviation.

**Table S1.** Gene-specific primer sequences for real-time quantitative PCR used in human and mice retinal biopsies.

| ***Gen*** |  | **Human** | **Mouse** |
| --- | --- | --- | --- |
| *BAX* | Forward (5' – 3') | ATCCAGGATCGAGCAGGGCG | CCCGAGAGGTCTTCTTCC |
|  | Reverse (5' – 3') | ACTCGCTCAGCTTCTTGGTG | GCCTTGAGCACCAGTTTG |
| *BCL2* | Forward (5' – 3') | GGGGTCATGTGTGTGGAGAG | TTCAGGGATGGGGTGAACTG |
|  | Reverse (5' – 3') | ACCTACCCAGCCTCCGTTAT | CACAGGGCGATGTTGT |
| *CASP3* | Forward (5' – 3') | GTTGATGATGACATGGCGTG | GTGGACTCTGGGATCTATCT |
|  | Reverse (5' – 3') | TCAAGCTTGTCGGCATACTG | CCATGAATGTCTCTCTGAGG |
| *CASP8* | Forward (5' – 3') | CTTGGATGCAGGGGCTTTGACC | CGGGAAGACATAACCCAACT |
|  | Reverse (5' – 3') | GTTCACTTCAGTCAGGATGG | GTGGGATAGGATACAGCAGA |
| *CASP9* | Forward (5' – 3') | AGGTTCTCAGACCGGAAACA | AGTTCCCGGGTGCTGTCTAT |
|  | Reverse (5' – 3') | TTGATAATGAGGCAGTGGCC | GCCATGGTCTTTCTGCTCAC |
| *ATG7* | Forward (5' – 3') | CCCAGCTATTGGAACACTGT | CTCCCTGGTCCTGATCTCCA |
|  | Reverse (5' – 3') | GTTTTCAAGAGCAGTGCCTG | GTGCACATGGAAGGACCTGA |
| *BECLIN1* | Forward (5' – 3') | AGGGATGGAAGGGTCTAAG | GTGCGCTACGCCCAGATC |
|  | Reverse (5' – 3') | GGGCTGTGGTAAGTAATGG | GATGTGGAAGGTGGCATTGAA |
| *LC3B* | Forward (5' – 3') | TAGGCCTGAGTTGTGAAGCG | GCGACTGGAGAGCTGTTTCT |
|  | Reverse (5' – 3') | TCTCACACAGCCCGTTTACC | AACCACATCCTAAGGCCAGC |
| *MTOR* | Forward (5' – 3') | AGTGGACCAGTGGAAACAGG | CTGCAGAATCGTCAAGGGGT |
|  | Reverse (5' – 3') | TTCAGCGATGTCTTGTGAGG | ATCTCCCTGGCTGCTCCTTA |
| *SQSTM1* | Forward (5' – 3') | TAGTCGTGTGGGTCGAGGAT | GGTGTGGTTCGCAGTACTGA |
|  | Reverse (5' – 3') | AAGACCTCATGGGACGCAAG | AGCATCCTGCCAAGCTGAAT |
| *IL1* | Forward (5' – 3') | GCCTTGAGCCAACAAATGCA | ACAAACCACCCGTTTCACCT |
|  | Reverse (5' – 3') | AAGAGCTGTGTTCCTTCCGG | ATGGGTGGAGGGTTCACTCT |
| *IL6* | Forward (5' – 3') | ACTCACCTCTTCAGAACGAATTG | GAGGATACCACTCCCAACAGACC |
|  | Reverse (5' – 3') | CCATCTTTGGAAGGTTCAGGTTG | AAGTGCATCATCGTTGTTCATACA |
| *IL10* | Forward (5' – 3') | GCCTAACATGCTTCGAGATC | GCTCTTACTGACTGGCATGAG |
|  | Reverse (5' – 3') | CTCATGGCTTTGTAGATGCC | CGCAGCTCTAGGAGCATGTG |
| *TGFB1* | Forward (5' – 3') | GACTTTTCCCCAGACCTCGG | CCTGGGTTGGAAGTGGATCC |
|  | Reverse (5' – 3') | ATAGGGGATCTGTGGCAGGT | GGTTGTAGAGGGCAAGGACC |
| *TNFA* | Forward (5' – 3') | CAGAGGGAAGAGTTCCCCAG | ACCCTCACACTCAGATCATCTTC |
|  | Reverse (5' – 3') | CCTTGGTCTGGTAGGAGACG | TGGTGGTTTGCTACGACGT |
| *cFOS* | Forward (5' – 3') | GCGGAGGGACAAGATCAACA | TACTACCATTCCCCAGCCGA |
|  | Reverse (5' – 3') | CTCCCGTCTTGCTGTTGTCT | GCTGTCACCGTGGGGATAAA |
| *NFKB* | Forward (5' – 3') | TGTGCTTCGAGTGACTGACC | GGAAGTGCTGCTGGGTAGTT |
|  | Reverse (5' – 3') | TCACCCCACATCACTGAACG | CTCATGCCCAAACTGGTTGC |
| *S100b* | Forward (5' – 3') | GGCCTGTGTGTAAGCTGACT | AGGGTGTAGGCGATCAGTCT |
|  | Reverse (5' – 3') | CCTCCGGGTTAGGGTCTACA | GTTGTGTTCAAGCAGCCTGG |
| *GFAP* | Forward (5' – 3') | GCACGCAGTATGAGGCAATG | GTTGTGTTCAAGCAGCCTGG |
|  | Reverse (5' – 3') | TAGTCGTTGGCTTCGTGCTT | AACTGAGCGGACACTGTCTG |
| *GAPDH* | Forward (5' – 3') | TGCACCACCAACTGCTTAGC | |
|  | Reverse (5' – 3') | CACCACCTTCTTGATGTCATCA | |

**Table S2.** Enzyme-linked immunosorbent Assay (ELISA) kits used in human and mice retinal biopsies.

| **Protein** | **Commercial Kits for human retinal biopsies** | **Commercial Kits for mice retinal biopsies** |
| --- | --- | --- |
| Casp3 | SEA626Hu, Cloud-Clone Corp | SEA626Mu, Cloud-Clone Corp |
| IL6 | ELH-IL6-CL, Ray Biotech | E-EL-M0044, Elabscience |
| TGF-β1 | EH0287, Fine Test | E-EL-0162, Elabscience |
| P62 | EH10842, Fine Test | ADI-900-212-0001, Enzo |
| cFOS | ab264626, Abcam | ab303739, Abcam |
| NF-kB-p65 | ab176648, Abcam | ab176648, Abcam |
